# Supplementary material for: Genetic determinants of host- and virus-derived insertions for hepatitis E virus replication
Source: Nat Commun. 2024 Jun 6;15:4855. doi: 10.1038/s41467-024-49219-8 (PMC11156872; doi:10.1038/s41467-024-49219-8)
Supplement: Supplementary file 1 — Supplementary Information [file 41467_2024_49219_MOESM1_ESM.pdf]

Supplementary Information to

Genetic determinants of host-and virus-derived insertions for hepatitis E virus replication

Michael Hermann Wißing, Toni Luise Meister, Maximilian Klaus Nocke, André Gömer, Mejrema Masovic, Leonard Knegendorf, Yannick Brüggemann, Verian Bader, Anindya Siddharta, Claus-Thomas Bock, Alexander Ploss, Scott P Kenney, Konstanze F Winklhofer, Patrick Behrendt, Heiner Wedemeyer, Eike Steinmann[#], Daniel Todt[#]

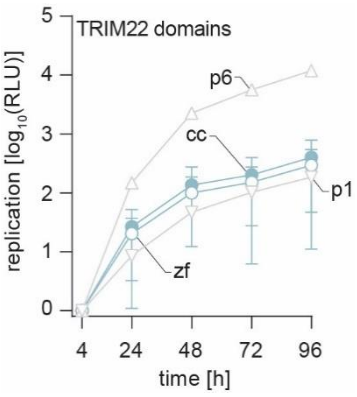

Supplementary Fig. 1: In vitro characterization of alternative domain gene snippets.

Replication kinetics of constructs as described in Fig. 2 with in vivo identified TRIM22 sequence snippets replaced with sequences derived from TRIM22 zinc finger domain (zf, GenBank accession number PP408296) or coiled coil region (cc, GenBank accession number PP408297). Kernow C1 p6 (p6) and Kernow C1 p1 (p1) shown as references. Plotted is the time post electroporation as well as relative light units (RLU) normalized to the four-hour value, mean ± SD.

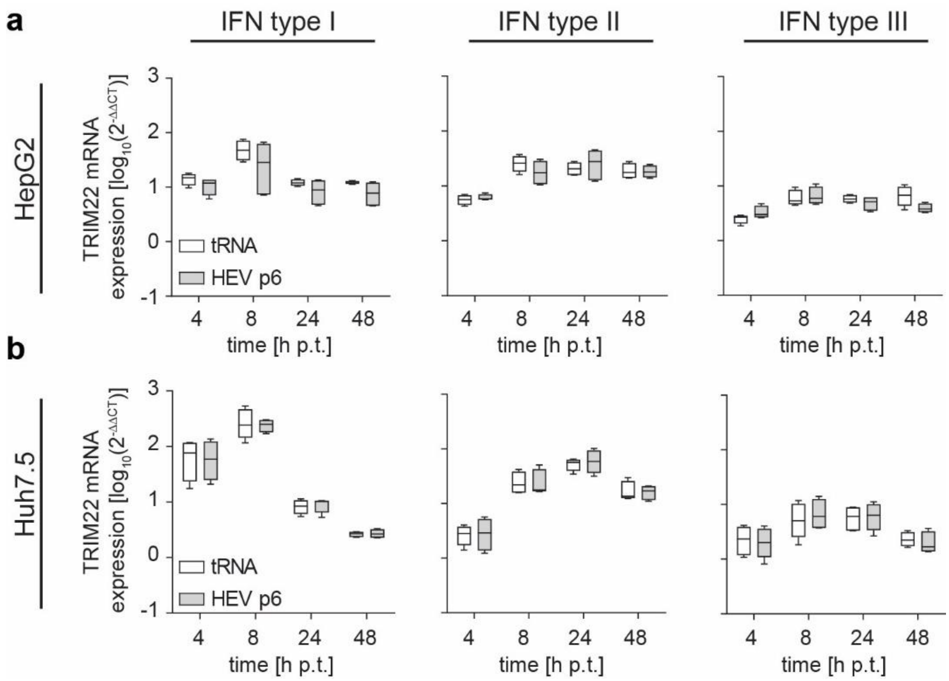

Supplementary Fig. 2: Impaired downregulation of IFN induced TRIM22 expression after HEV p6 genome transfection.

a HepG2 or **b** Huh7.5 cells were electroporated with either tRNA (control) or Kernow-C1-p6 (p6) and treated with 40 fM of different types of interferon (IFN) for indicated time periods. RNA was harvested and TRIM22 expression was measured via qRT-PCR. Depicted are ΔΔCT values of three independent replicates as boxplots with median and, 25% quartiles and range.

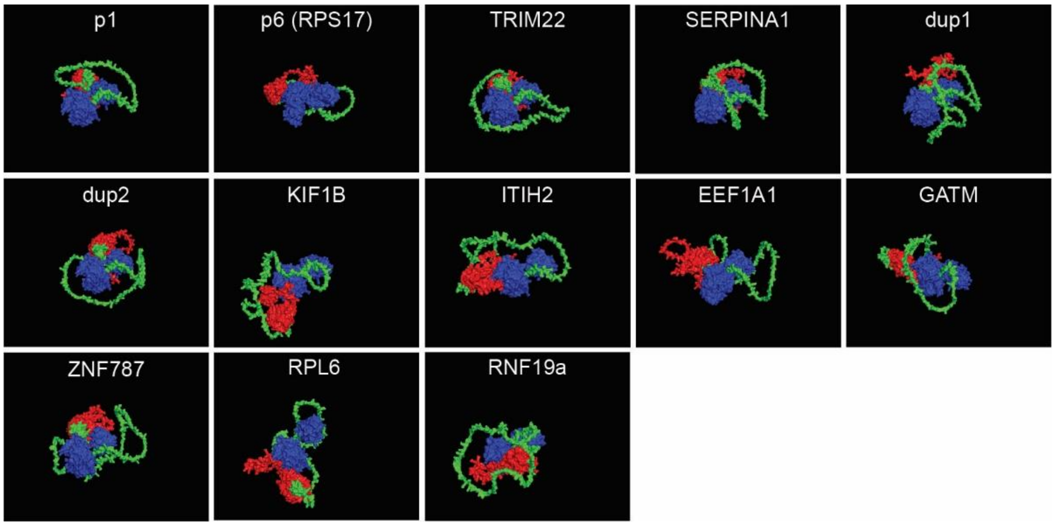

Supplementary Fig. 3: AlphaFold models of insertion containing HVRs.

The protein structure of insertion containing partial ORF1 sequences was predicted. Therefore, the sequence starting at the 5'-end of the PCP domain until the 3'-end of the helicase was predicted via AlphaFold2. The generated PDB files were analyzed in PyMol. Depicted are the surface models of the PCP domain (red), the HVR (green) and the X-domain with helicase (blue). The 3D structure of the X-Domain with helicase were conserved and used to superimpose all structures. See **Supplementary Movies 1-3**.

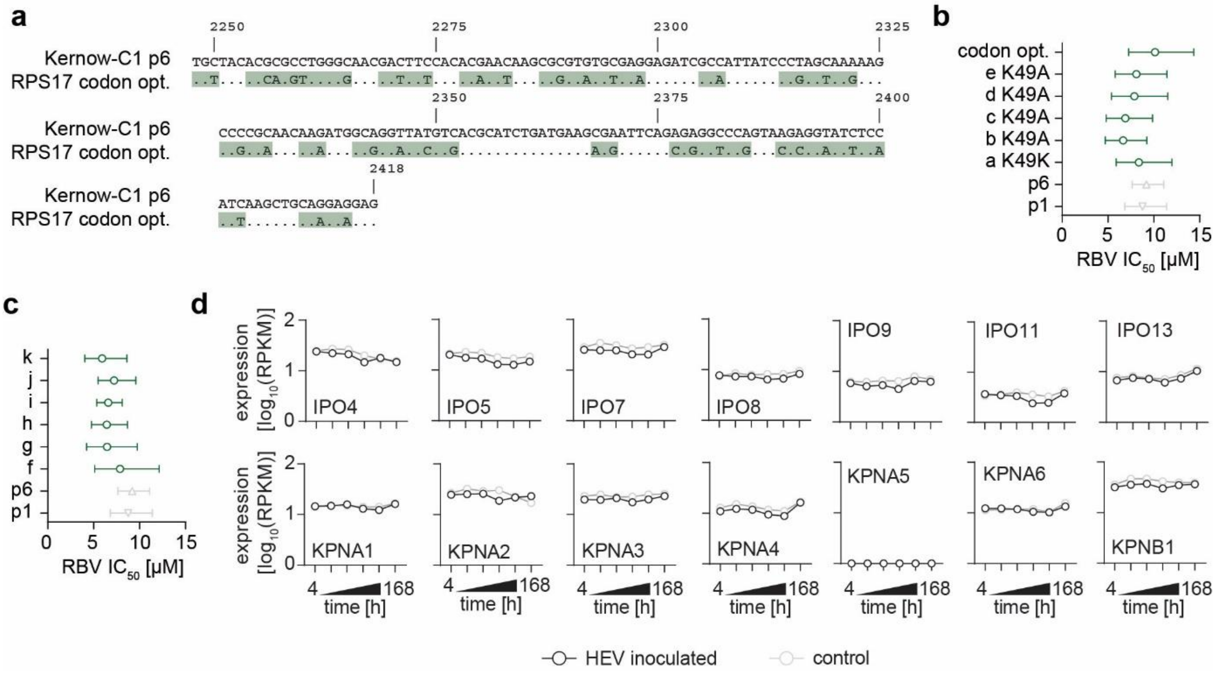

Supplementary Fig. 4: Nucleotide alignment of codon optimized RPS17 insertion and RBV susceptibility of NLS constructs.

a Nucleotide alignment between the RPS17 insertion of the Kernow-C1-p6 strain and the codon optimized version are shown. Dots represent identity, while mismatches are depicted by the used nucleotide in the codon optimized version. Green boxes highlight altered codons. The coordinates are based on the Kernow-C1-p6 ORF1. **b, c** The replicon was used to analyse the ribavirin (RBV) sensitivity of the NLS mutants by treating the cells for five days post electroporation with RBV concentrations ranging from 0.19 µM to 100 µM. A non-linear regression and the IC_{50} values were calculated using GraphPad Prism. Depicted are the means with confidence intervals (CI 95%) of at least three independent replicates. **d** Expression of transcript encoding importin subunits was analyzed in non-infected as well as Kernow-C1-p6 infected primary human hepatocytes (PHH). Depicted is the expression as RPKM values over time for importin subunits (IPO4-13, upper panel) and karyopherin-α proteins (KPNA1-6) as well as karyopherin-β protein (KPNB1).

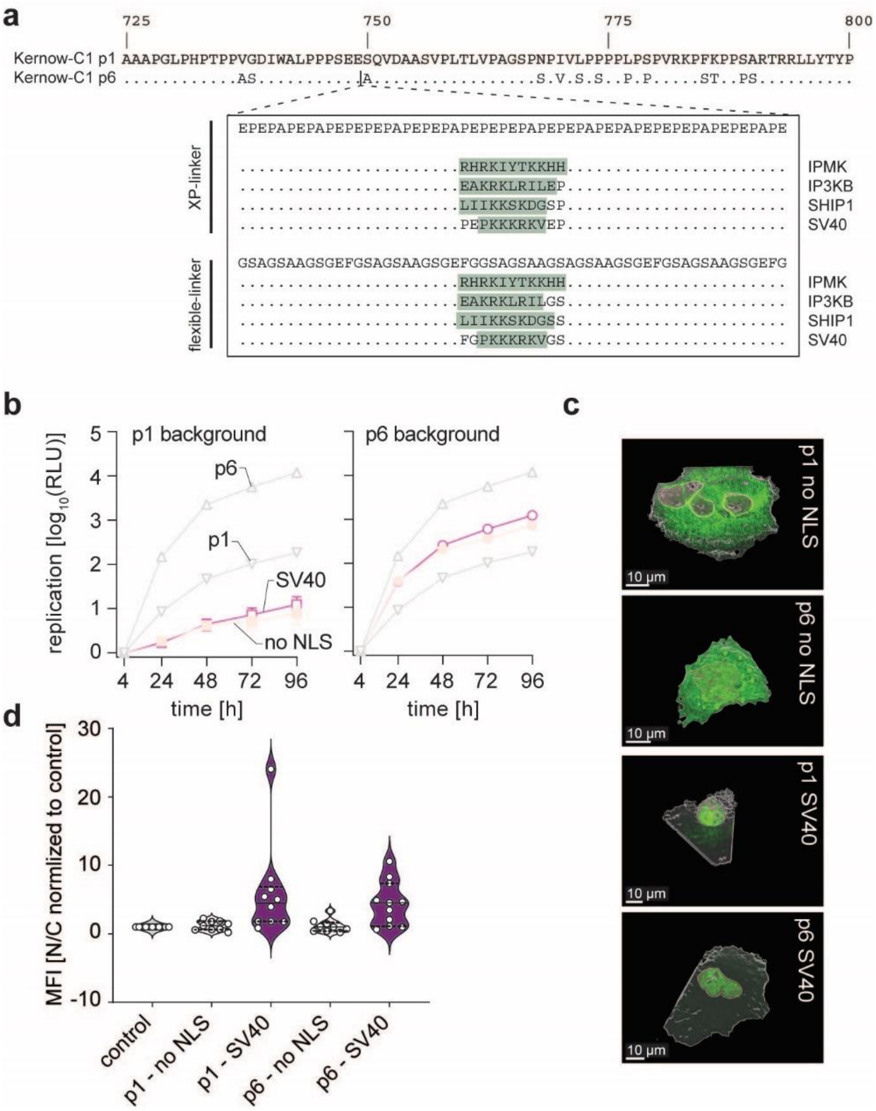

Supplementary Fig. 5: Characterization of HVR constructs with artificial insertions.

a The amino acid sequence of the artificial insertions and their NLS sequences (green) are depicted. The arrow indicates the insertion site in the Kernow-C1-p1 and Kernow-C1-p6 construct. The RPS17 insertion of the p6 construct was substituted with the artificial insertions. The green boxes indicate the NLS sequence which are ranked in increasing strength IPKM<IP3KB<SHIp1<SV40. **b** Replication kinetics of constructs with shuffled RPS17 sequences (GenBank accession numbers PP408298-PP408301). Kernow-C1-p6 (p6) and Kernow-C1-p1 (p1) shown as references. Plotted is the time post electroporation as well as relative light units (RLU) normalized to the four-hour value, mean ± SD. **c** Huh7 cells were transfected with plasmids encoding a triple eYFP in tandem with the shuffled RPS17 sequences and flanking regions of the constructs Kernow-C1-p1 (p1), Kernow-C1-p6 (p6), cells were

fixed after 16-20 hours and the nucleus as well as the cell membrane were stained via immunofluorescence. The cells were imaged in 3D using a Zeiss Elyra 7. The cell and nuclear surface were reconstructed using Imaris 10.0.1 and the mean fluorescence intensity (MFI) for eYFP was measured for each compartment. Shown are example cells in 3D. eYFP is shown in green, the cell surface is depicted in white while the nuclear surface is depicted in red. See **Supplementary Movies 27-30**. **d** The eYFP MFI was measured for each compartment for ten cells per construct. The ratio of nuclear (N) to cytoplasmic (C) MFI was calculated. Depicted is the range of individual data points as violin plot with straight line as median and dashed lines as quartiles. Single outliers were removed after applying ROUT outlier test (Q=1).

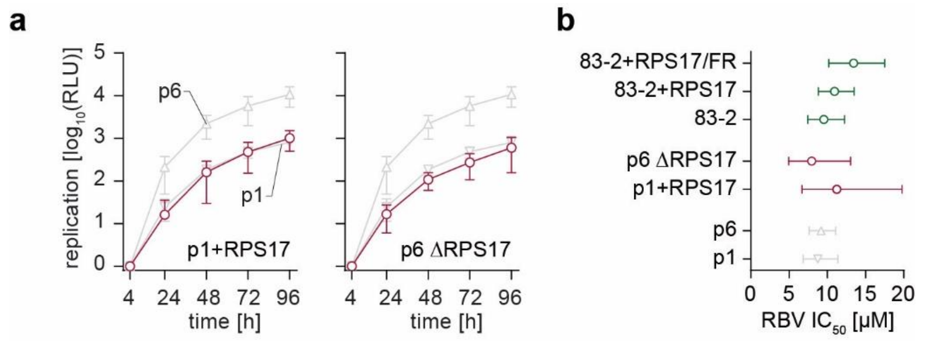

Supplementary Fig. 6: Impact of deleting and inserting the RPS17 insertion on HEV replication and RBV sensitivity.

a The RPS17 insertion was cloned into the corresponding site of Kernow-C1-p1 and deleted from Kernow-C1-p6. Replication kinetics were measured with p1 and p6 as reference (both grey) while constructs of interest are depicted in red. Plotted are relative light units (RLU) normalized to the four-hour value over time (hours post electroporation). **b** The replicon was used to analyse the ribavirin (RBV) sensitivity of p1 and 83-2 mutants by treating the cells for five days post electroporation with RBV concentrations ranging from 0.19 µM to 100 µM. A non-linear regression and the IC_{50} values were calculated using GraphPad Prism. Depicted are the means with confidence intervals (CI 95%) of at least three independent replicates.
