# Supplementary material for: Genetic determinants of host- and virus-derived insertions for hepatitis E virus replication
Source: Nat Commun. 2024 Jun 6;15:4855. doi: 10.1038/s41467-024-49219-8 (PMC11156872; doi:10.1038/s41467-024-49219-8)
Supplement: Supplementary file 3 — Description of Additional Supplementary Files [file 41467_2024_49219_MOESM3_ESM.pdf]

Supplementary Information to

Genetic determinants of host-and virus-derived insertions for hepatitis E virus replication

Michael Hermann Wißing[1], Toni Luise Meister[1], Maximilian Klaus Nocke[1,3], André Gömer[1], Mejrema Masovic[1], Leonard Knegendorf[2,4], Yannick Brüggemann[1], Verian Bader[5,6], Anindya Siddharta[2], Claus-Thomas Bock[7], Alexander Ploss[8], Scott P Kenney[9], Konstanze F Winklhofer[5,10], Patrick Behrendt[2,11,12], Heiner Wedemeyer[11,12,13], Eike Steinmann[1,14#], Daniel Todt[1,3#]

File Name: Supplementary Movie 1:

Description: AlphaFold models of HVR insertion identified in this study.

The protein structure of partial ORF1 including HVR insertions RPS17 (p6), TRIM22, SERPINA1, duplication 1 and duplication 2, as well as Kernow-C1-p1 without insertion, was predicted via AlphaFold2. Generated PDB files were analysed in PyMol. Depicted are the surface models of the PCP domain (red), the HVR (green) and the X-domain with helicase (blue). The 3D structure of the X-Domain with helicase were conserved and used to superimpose all structures.

File Name: Supplementary Movie 2:

Description: AlphaFold models of HVR insertion previously published.

The protein structure of partial ORF1 including HVR insertions EEF1A1, ZNF787, GATM, ITIH2 or RPL6, as well as Kernow-C1-p1 without insertion, was predicted via AlphaFold2. Generated PDB files were analysed in PyMol. Depicted are the surface models of the PCP domain (red), the HVR (green) and the X-domain with helicase (blue). The 3D structure of the X-Domain with helicase were conserved and used to superimpose all structures.

File Name: Supplementary Movie 3:

Description: AlphaFold models of HVR insertion previously published.

The protein structure of partial ORF1 including HVR insertions RNF19a, KIF1B, as well as Kernow-C1-p1 without insertion, was predicted via AlphaFold2. Generated PDB files were analysed in PyMol. Depicted are the surface models of the PCP domain (red), the HVR (green) and the X-domain with helicase (blue). The 3D structure of the X-Domain with helicase were conserved and used to superimpose all structures.

File Name: Supplementary Movie 4:

Description: Nuclear localization of eYFP construct control.

Huh7 cells were transfected with plasmids encoding a triple eYFP alone (control). Cells were fixed after 16-20 hours and the nucleus as well as the cell membrane were stained via immunofluorescence. The

cells were imaged in 3D using a Zeiss Elyra 7. The cell and nuclear surface were reconstructed using Imaris 10.0.1 and the mean fluorescence intensity (MFI) for eYFP was measured for each compartment. Shown are example cells in 3D. eYFP is shown in green, the cell surface is depicted in red while the nuclear surface is depicted in light blue.

File Name: Supplementary Movie 5:

Description: Nuclear localization of eYFP construct p1.

Huh7 cells were transfected with plasmids encoding a triple eYFP in tandem with the HVR of the construct Kernow-C1-p1 (p1). Cells were fixed after 16-20 hours and the nucleus as well as the cell membrane were stained via immunofluorescence. The cells were imaged in 3D using a Zeiss Elyra 7. The cell and nuclear surface were reconstructed using Imaris 10.0.1 and the mean fluorescence intensity (MFI) for eYFP was measured for each compartment. Shown are example cells in 3D. eYFP is shown in green, the cell surface is depicted in red while the nuclear surface is depicted in light blue.

File Name: Supplementary Movie 6:

Description: Nuclear localization of eYFP construct p6.

Huh7 cells were transfected with plasmids encoding a triple eYFP in tandem with the HVR of the construct Kernow-C1-p6 (p6) containing RPS17 insertion. Cells were fixed after 16-20 hours and the nucleus as well as the cell membrane were stained via immunofluorescence. The cells were imaged in 3D using a Zeiss Elyra 7. The cell and nuclear surface were reconstructed using Imaris 10.0.1 and the mean fluorescence intensity (MFI) for eYFP was measured for each compartment. Shown are example cells in 3D. eYFP is shown in green, the cell surface is depicted in red while the nuclear surface is depicted in light blue.

File Name: Supplementary Movie 7:

Description: Nuclear localization of eYFP construct lysine mutant.

Huh7 cells were transfected with plasmids encoding a triple eYFP in tandem with the HVR of the construct Kernow-C1-p6 (p6) containing lysine mutants of the RPS17 insertion. Cells were fixed after 16-20 hours and the nucleus as well as the cell membrane were stained via immunofluorescence. The cells were imaged in 3D using a Zeiss Elyra 7. The cell and nuclear surface were reconstructed using Imaris 10.0.1 and the mean fluorescence intensity (MFI) for eYFP was measured for each compartment. Shown are example cells in 3D. eYFP is shown in green, the cell surface is depicted in red while the nuclear surface is depicted in light blue.

File Name: Supplementary Movie 8:

Description: Nuclear localization of eYFP SERPINA1.1 construct.

Huh7 cells were transfected with plasmids encoding a triple eYFP in tandem with the HVR of the construct Kernow-C1-p6 (p6) containing the SERPINA1.1 insertion. Cells were fixed after 16-20 hours and the nucleus as well as the cell membrane were stained via immunofluorescence. The cells were imaged in 3D using a Zeiss Elyra 7. The cell and nuclear surface were reconstructed using Imaris 10.0.1 and the mean fluorescence intensity (MFI) for eYFP was measured for each compartment. Shown are example cells in 3D. eYFP is shown in green, the cell surface is depicted in red while the nuclear surface is depicted in light blue.

File Name: Supplementary Movie 9:

Description: Nuclear localization of eYFP SERPINA1.2 construct.

Huh7 cells were transfected with plasmids encoding a triple eYFP in tandem with the HVR of the construct Kernow-C1-p6 (p6) containing the SERPINA1.2 insertion. Cells were fixed after 16-20 hours and the nucleus as well as the cell membrane were stained via immunofluorescence. The cells were imaged in 3D using a Zeiss Elyra 7. The cell and nuclear surface were reconstructed using Imaris 10.0.1 and the mean fluorescence intensity (MFI) for eYFP was measured for each compartment. Shown are

example cells in 3D. eYFP is shown in green, the cell surface is depicted in red while the nuclear surface is depicted in light blue.

File Name: Supplementary Movie 10:

Description: Nuclear localization of eYFP cons.TRIM22 construct.

Huh7 cells were transfected with plasmids encoding a triple eYFP in tandem with the HVR of the construct Kernow-C1-p6 (p6) containing the cons.TRIM22 insertion. Cells were fixed after 16-20 hours and the nucleus as well as the cell membrane were stained via immunofluorescence. The cells were imaged in 3D using a Zeiss Elyra 7. The cell and nuclear surface were reconstructed using Imaris 10.0.1 and the mean fluorescence intensity (MFI) for eYFP was measured for each compartment. Shown are example cells in 3D. eYFP is shown in green, the cell surface is depicted in red while the nuclear surface is depicted in light blue.

File Name: Supplementary Movie 11:

Description: Nuclear localization of eYFP h.TRIM22 construct.

Huh7 cells were transfected with plasmids encoding a triple eYFP in tandem with the HVR of the construct Kernow-C1-p6 (p6) containing the h.TRIM22 insertion. Cells were fixed after 16-20 hours and the nucleus as well as the cell membrane were stained via immunofluorescence. The cells were imaged in 3D using a Zeiss Elyra 7. The cell and nuclear surface were reconstructed using Imaris 10.0.1 and the mean fluorescence intensity (MFI) for eYFP was measured for each compartment. Shown are example cells in 3D. eYFP is shown in green, the cell surface is depicted in red while the nuclear surface is depicted in light blue.

File Name: Supplementary Movie 12:

Description: Nuclear localization of eYFP dup1 construct.

Huh7 cells were transfected with plasmids encoding a triple eYFP in tandem with the HVR of the construct Kernow-C1-p6 (p6) containing the dup1 insertion. Cells were fixed after 16-20 hours and the

nucleus as well as the cell membrane were stained via immunofluorescence. The cells were imaged in 3D using a Zeiss Elyra 7. The cell and nuclear surface were reconstructed using Imaris 10.0.1 and the mean fluorescence intensity (MFI) for eYFP was measured for each compartment. Shown are example cells in 3D. eYFP is shown in green, the cell surface is depicted in red while the nuclear surface is depicted in light blue.

File Name: Supplementary Movie 13:

Description: Nuclear localization of eYFP dup2 construct.

Huh7 cells were transfected with plasmids encoding a triple eYFP in tandem with the HVR of the construct Kernow-C1-p6 (p6) containing the dup2 insertion. Cells were fixed after 16-20 hours and the nucleus as well as the cell membrane were stained via immunofluorescence. The cells were imaged in 3D using a Zeiss Elyra 7. The cell and nuclear surface were reconstructed using Imaris 10.0.1 and the mean fluorescence intensity (MFI) for eYFP was measured for each compartment. Shown are example cells in 3D. eYFP is shown in green, the cell surface is depicted in red while the nuclear surface is depicted in light blue.

File Name: Supplementary Movie 14:

Description: AlphaFold models of Kernow-C1-p1 HVR with artificial flexible linker.

The protein structure of partial ORF1 including Kernow-C1-p1 HVR with artificial flexible linker, as well as Kernow-C1-p6 (RPS17 insertion), was predicted via AlphaFold2. Generated PDB files were analysed in PyMol. Depicted are the surface models of the PCP domain (red), the HVR (green) and the X-domain with helicase (blue). The 3D structure of the X-Domain with helicase were conserved and used to superimpose all structures.

File Name: Supplementary Movie 15:

Description: AlphaFold models of Kernow-C1-p6 HVR with artificial flexible linker.

The protein structure of partial ORF1 including Kernow-C1-p6 HVR (RPS17 insertion) with artificial flexible linker, as well as Kernow-C1-p6 (RPS17 only), was predicted via AlphaFold2. Generated PDB files were analysed in PyMol. Depicted are the surface models of the PCP domain (red), the HVR (green) and the X-domain with helicase (blue). The 3D structure of the X-Domain with helicase were conserved and used to superimpose all structures.

File Name: Supplementary Movie 16:

Description: AlphaFold models of Kernow-C1-p1 HVR with artificial rigid linker.

The protein structure of partial ORF1 including Kernow-C1-p1 HVR with artificial rigid linker, as well as Kernow-C1-p6 (RPS17 insertion), was predicted via AlphaFold2. Generated PDB files were analysed in PyMol. Depicted are the surface models of the PCP domain (red), the HVR (green) and the X-domain with helicase (blue). The 3D structure of the X-Domain with helicase were conserved and used to superimpose all structures.

File Name: Supplementary Movie 17:

Description: AlphaFold models of Kernow-C1-p6 HVR with rigid flexible linker.

The protein structure of partial ORF1 including Kernow-C1-p6 HVR (RPS17 insertion) with artificial rigid linker, as well as Kernow-C1-p6 (RPS17 only), was predicted via AlphaFold2. Generated PDB files were analysed in PyMol. Depicted are the surface models of the PCP domain (red), the HVR (green) and the X-domain with helicase (blue). The 3D structure of the X-Domain with helicase were conserved and used to superimpose all structures.

File Name: Supplementary Movie 18:

Description: Nuclear localization of eYFP rigid linker p1 construct.

Huh7 cells were transfected with plasmids encoding a triple eYFP in tandem with a rigid linker inserted into the p1 backbone. Cells were fixed after 16-20 hours and the nucleus as well as the cell membrane were stained via immunofluorescence. The cells were imaged in 3D using a Zeiss Elyra 7. The cell and nuclear surface were reconstructed using Imaris 10.0.1 and the mean fluorescence intensity (MFI) for eYFP was measured for each compartment. Shown are example cells in 3D. eYFP is shown in green, the cell surface is depicted in red while the nuclear surface is depicted in light blue.

File Name: Supplementary Movie 19:

Description: Nuclear localization of eYFP rigid linker plus SV40 NLS p1 construct.

Huh7 cells were transfected with plasmids encoding a triple eYFP in tandem with a SV40 NLS flanked by a rigid linker inserted into the p1 backbone. Cells were fixed after 16-20 hours and the nucleus as well as the cell membrane were stained via immunofluorescence. The cells were imaged in 3D using a Zeiss Elyra 7. The cell and nuclear surface were reconstructed using Imaris 10.0.1 and the mean fluorescence intensity (MFI) for eYFP was measured for each compartment. Shown are example cells in 3D. eYFP is shown in green, the cell surface is depicted in red while the nuclear surface is depicted in light blue.

File Name: Supplementary Movie 20:

Description: Nuclear localization of eYFP rigid linker p6 construct.

Huh7 cells were transfected with plasmids encoding a triple eYFP in tandem with a rigid linker inserted into the p6 backbone replacing the RSP17 snippet. Cells were fixed after 16-20 hours and the nucleus as well as the cell membrane were stained via immunofluorescence. The cells were imaged in 3D using a Zeiss Elyra 7. The cell and nuclear surface were reconstructed using Imaris 10.0.1 and the mean fluorescence intensity (MFI) for eYFP was measured for each compartment. Shown are example cells

in 3D. eYFP is shown in green, the cell surface is depicted in red while the nuclear surface is depicted in light blue.

File Name: Supplementary Movie 21:

Description: Nuclear localization of eYFP rigid linker plus SV40 NLS p6 construct.

Huh7 cells were transfected with plasmids encoding a triple eYFP in tandem with a SV40 NLS flanked by a rigid linker inserted into the p6 backbone replacing the RSP17 snippet. Cells were fixed after 16-20 hours and the nucleus as well as the cell membrane were stained via immunofluorescence. The cells were imaged in 3D using a Zeiss Elyra 7. The cell and nuclear surface were reconstructed using Imaris 10.0.1 and the mean fluorescence intensity (MFI) for eYFP was measured for each compartment. Shown are example cells in 3D. eYFP is shown in green, the cell surface is depicted in red while the nuclear surface is depicted in light blue.

File Name: Supplementary Movie 22:

Description: Nuclear localization of eYFP flexible linker p1 construct.

Huh7 cells were transfected with plasmids encoding a triple eYFP in tandem with a flexible linker inserted into the p1 backbone. Cells were fixed after 16-20 hours and the nucleus as well as the cell membrane were stained via immunofluorescence. The cells were imaged in 3D using a Zeiss Elyra 7. The cell and nuclear surface were reconstructed using Imaris 10.0.1 and the mean fluorescence intensity (MFI) for eYFP was measured for each compartment. Shown are example cells in 3D. eYFP is shown in green, the cell surface is depicted in red while the nuclear surface is depicted in light blue.

File Name: Supplementary Movie 23:

Description: Nuclear localization of eYFP flexible linker plus SV40 NLS p1 construct.

Huh7 cells were transfected with plasmids encoding a triple eYFP in tandem with a SV40 NLS flanked by a flexible linker inserted into the p1 backbone. Cells were fixed after 16-20 hours and the nucleus as well as the cell membrane were stained via immunofluorescence. The cells were imaged in 3D using a

Zeiss Elyra 7. The cell and nuclear surface were reconstructed using Imaris 10.0.1 and the mean fluorescence intensity (MFI) for eYFP was measured for each compartment. Shown are example cells in 3D. eYFP is shown in green, the cell surface is depicted in red while the nuclear surface is depicted in light blue.

File Name: Supplementary Movie 24:

Description: Nuclear localization of eYFP flexible linker p6 construct.

Huh7 cells were transfected with plasmids encoding a triple eYFP in tandem with a flexible linker inserted into the p6 backbone replacing the RSP17 snippet. Cells were fixed after 16-20 hours and the nucleus as well as the cell membrane were stained via immunofluorescence. The cells were imaged in 3D using a Zeiss Elyra 7. The cell and nuclear surface were reconstructed using Imaris 10.0.1 and the mean fluorescence intensity (MFI) for eYFP was measured for each compartment. Shown are example cells in 3D. eYFP is shown in green, the cell surface is depicted in red while the nuclear surface is depicted in light blue.

File Name: Supplementary Movie 25:

Description: Nuclear localization of eYFP flexible linker plus SV40 NLS p6 construct.

Huh7 cells were transfected with plasmids encoding a triple eYFP in tandem with a SV40 NLS flanked by a flexible linker inserted into the p6 backbone replacing the RSP17 snippet. Cells were fixed after 16-20 hours and the nucleus as well as the cell membrane were stained via immunofluorescence. The cells were imaged in 3D using a Zeiss Elyra 7. The cell and nuclear surface were reconstructed using Imaris 10.0.1 and the mean fluorescence intensity (MFI) for eYFP was measured for each compartment. Shown are example cells in 3D. eYFP is shown in green, the cell surface is depicted in red while the nuclear surface is depicted in light blue.

File Name: Supplementary Movie 26:

Description: AlphaFold models of Kernow-C1-p6 HVR with shuffled RPS17 sequence.

The protein structure of partial ORF1 including the shuffled RS17 insertion with p1 or p6 flanking regions, respectively, containing a SV40 NLS or no NLS (GenBank accession numbers PP408298-PP408301) was predicted via AlphaFold2. Generated PDB files were analysed in PyMol. Depicted are the surface models of the PCP domain (red), the HVR (green) and the X-domain with helicase (blue). The 3D structure of the X-Domain with helicase were conserved and used to superimpose all structures.

File Name: Supplementary Movie 27:

Description: Nuclear localization of eYFP with shuffled RPS17 p1 construct.

Huh7 cells were transfected with plasmids encoding a triple eYFP in tandem with a shuffled RPS17 flanked by p1 HVR region (GenBank accession number PP408301). Cells were fixed after 16-20 hours and the nucleus as well as the cell membrane were stained via immunofluorescence. The cells were imaged in 3D using a Zeiss Elyra 7. The cell and nuclear surface were reconstructed using Imaris 10.0.1 and the mean fluorescence intensity (MFI) for eYFP was measured for each compartment. Shown are example cells in 3D. eYFP is shown in green, the cell surface is depicted in red while the nuclear surface is depicted in light blue.

File Name: Supplementary Movie 28:

Description: Nuclear localization of eYFP with shuffled RPS17 including a SV40 NLS p1 construct.

Huh7 cells were transfected with plasmids encoding a triple eYFP in tandem with a shuffled RPS17 and SV40 NLS flanked by p1 HVR region (GenBank accession number PP408300). Cells were fixed after 16-20 hours and the nucleus as well as the cell membrane were stained via immunofluorescence. The cells were imaged in 3D using a Zeiss Elyra 7. The cell and nuclear surface were reconstructed using Imaris 10.0.1 and the mean fluorescence intensity (MFI) for eYFP was measured for each compartment. Shown are example cells in 3D. eYFP is shown in green, the cell surface is depicted in red while the nuclear surface is depicted in light blue.

File Name: Supplementary Movie 29:

Description: Nuclear localization of eYFP with shuffled RPS17 p6 construct.

Huh7 cells were transfected with plasmids encoding a triple eYFP in tandem with a shuffled RPS17 flanked by p6 HVR region (GenBank accession number PP408298). Cells were fixed after 16-20 hours and the nucleus as well as the cell membrane were stained via immunofluorescence. The cells were imaged in 3D using a Zeiss Elyra 7. The cell and nuclear surface were reconstructed using Imaris 10.0.1 and the mean fluorescence intensity (MFI) for eYFP was measured for each compartment. Shown are example cells in 3D. eYFP is shown in green, the cell surface is depicted in red while the nuclear surface is depicted in light blue.

File Name: Supplementary Movie 30:

Description: Nuclear localization of eYFP with shuffled RPS17 including a SV40 NLS p6 construct.

Huh7 cells were transfected with plasmids encoding a triple eYFP in tandem with a shuffled RPS17 and SV40 NLS flanked by p6 HVR region (GenBank accession number PP408299). Cells were fixed after 16-20 hours and the nucleus as well as the cell membrane were stained via immunofluorescence. The cells were imaged in 3D using a Zeiss Elyra 7. The cell and nuclear surface were reconstructed using Imaris 10.0.1 and the mean fluorescence intensity (MFI) for eYFP was measured for each compartment. Shown are example cells in 3D. eYFP is shown in green, the cell surface is depicted in red while the nuclear surface is depicted in light blue.
