# Supplementary figures and images for: Genetic determinants of host- and virus-derived insertions for hepatitis E virus replication
Source: Nat Commun. 2024 Jun 6;15:4855. doi: 10.1038/s41467-024-49219-8 (PMC11156872; doi:10.1038/s41467-024-49219-8)

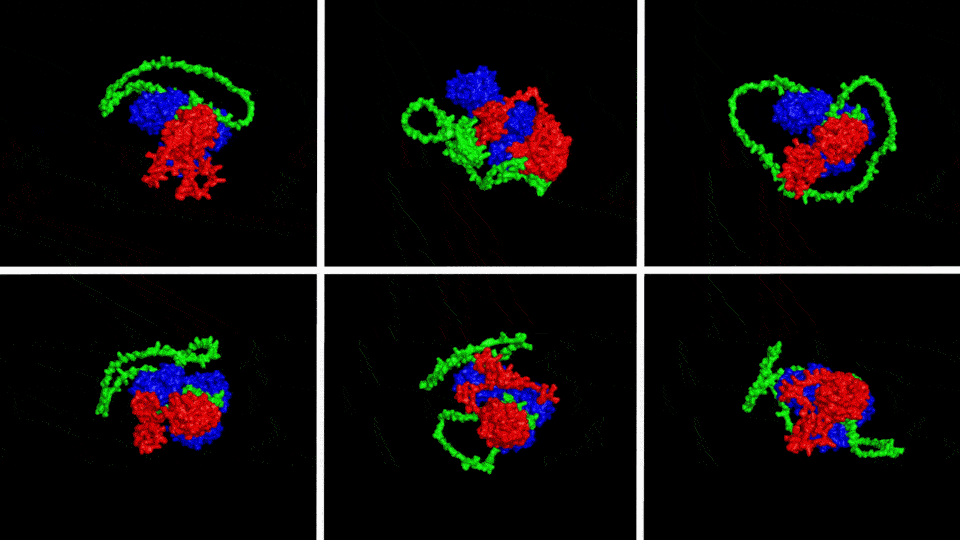

Supplement: Supplementary file 4 — supplementary movie files [file 41467_2024_49219_MOESM4_ESM.zip › Supplementary_Movie_1.gif]

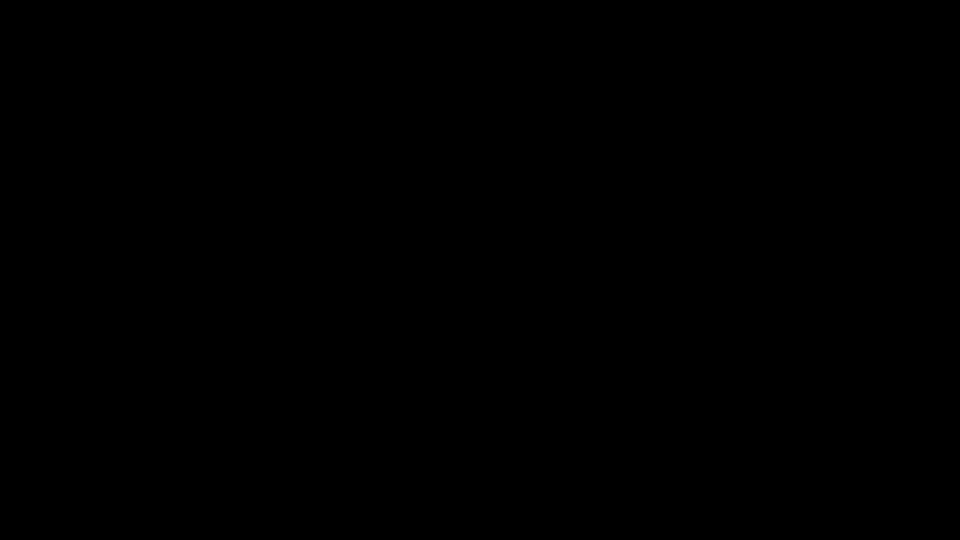

Supplement: Supplementary file 4 — supplementary movie files [file 41467_2024_49219_MOESM4_ESM.zip › Supplementary_Movie_14.gif]

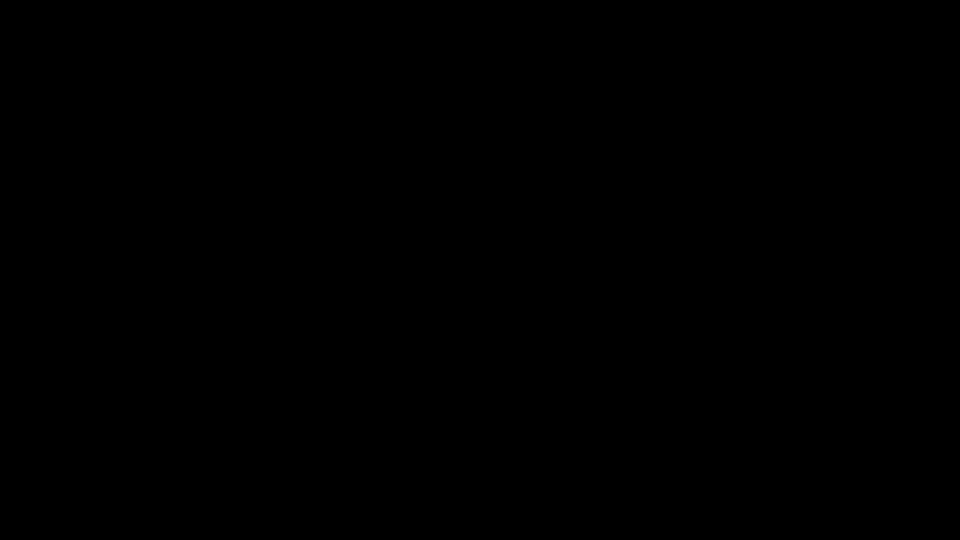

Supplement: Supplementary file 4 — supplementary movie files [file 41467_2024_49219_MOESM4_ESM.zip › Supplementary_Movie_15.gif]

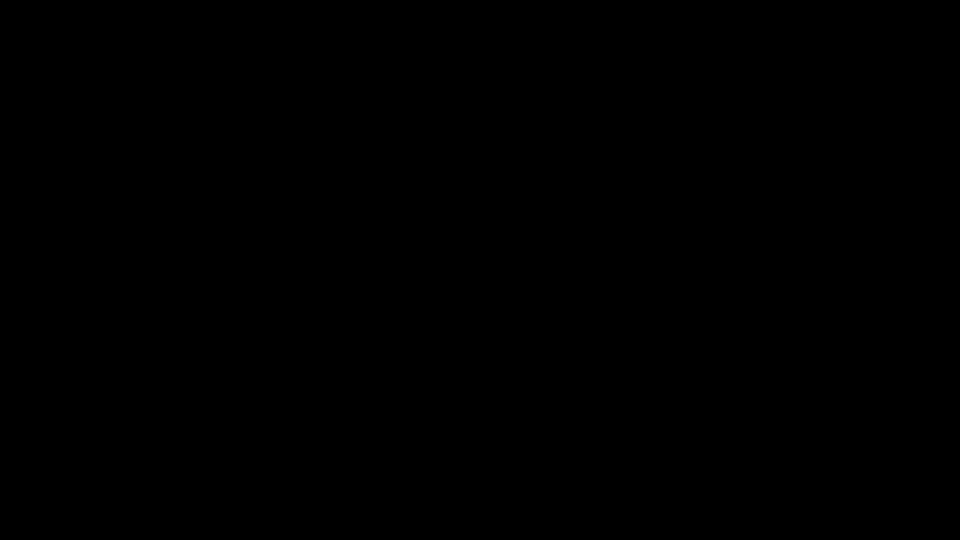

Supplement: Supplementary file 4 — supplementary movie files [file 41467_2024_49219_MOESM4_ESM.zip › Supplementary_Movie_16.gif]

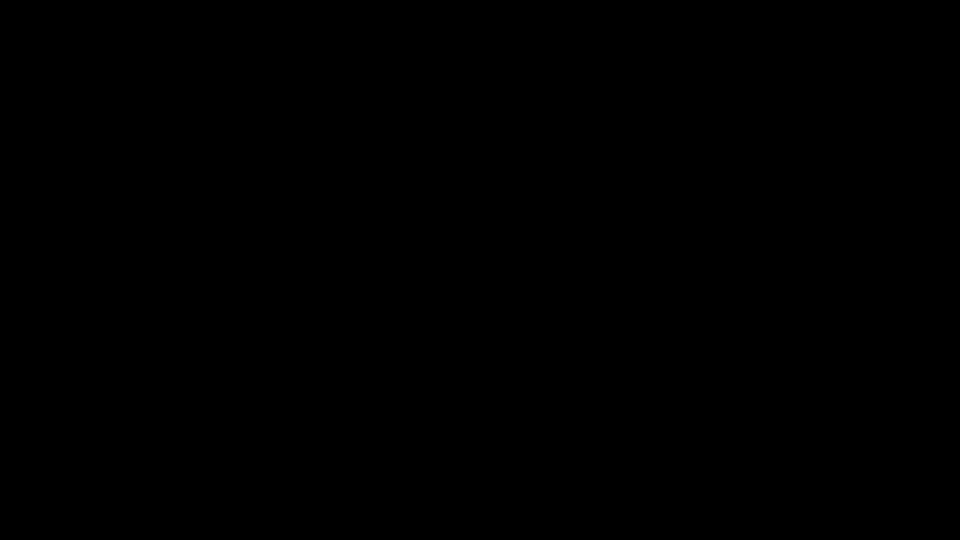

Supplement: Supplementary file 4 — supplementary movie files [file 41467_2024_49219_MOESM4_ESM.zip › Supplementary_Movie_17.gif]

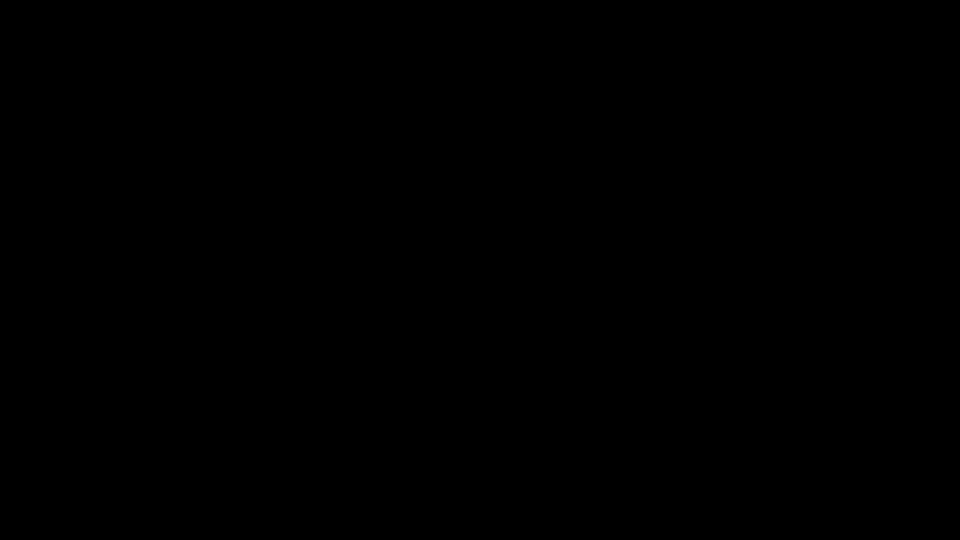

Supplement: Supplementary file 4 — supplementary movie files [file 41467_2024_49219_MOESM4_ESM.zip › Supplementary_Movie_2.gif]

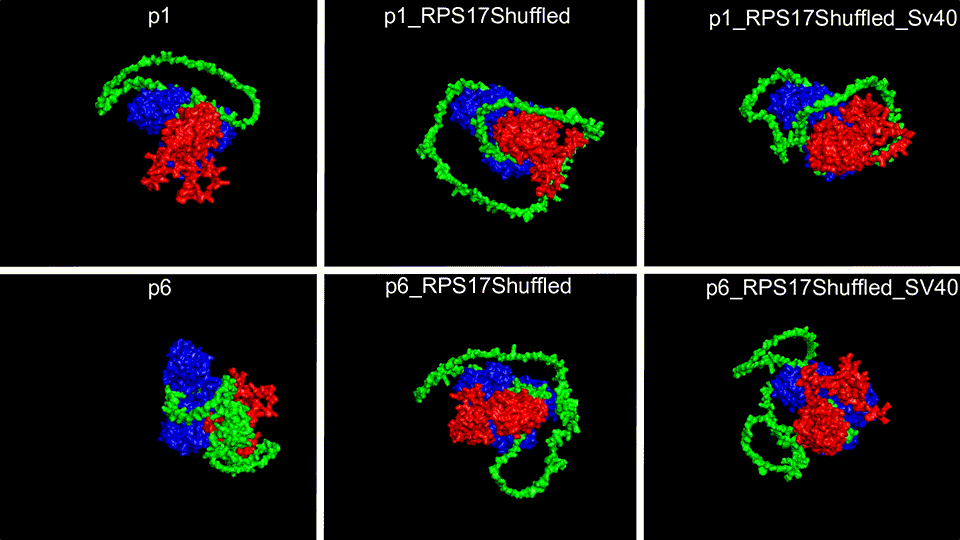

Supplement: Supplementary file 4 — supplementary movie files [file 41467_2024_49219_MOESM4_ESM.zip › Supplementary_Movie_26.gif]

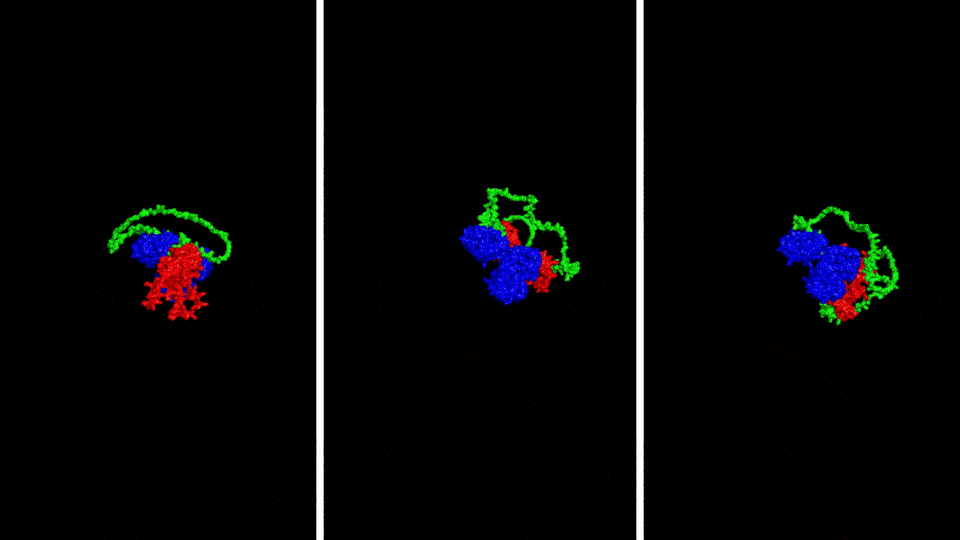

Supplement: Supplementary file 4 — supplementary movie files [file 41467_2024_49219_MOESM4_ESM.zip › Supplementary_Movie_3.gif]
